# Examining the efficacy of dance movement and music mixed treatment on social communication impairment in children with autism — Based on family parent-child situation

**DOI:** 10.3389/fpsyg.2022.937564

**Published:** 2022-08-08

**Authors:** Huiting Ren, Guanghao Ren, Yuqi Zhan, Zhichun Jia

**Affiliations:** ^1^School of Art, Hunan City University, Yiyang, China; ^2^Laboratory Department of Business Science, Sehan University, Mokpo, South Korea; ^3^Laboratory X, Central Hospital of Yiyang, Yiyang, China; ^4^The Second Ship Design Institute of Wuhan, Wuhan, China

**Keywords:** dance movement and music mixed therapy, ASD children, social communication impairments, parent-child family situation, music mixed therapy

## Abstract

Despite impairments in social communication in children with autism spectrum disorder (ASD), existing studies only examine the effects of either MT or DMT interventions. In the family setting, few studies have investigated interventions for social communication impairments in children with ASD. This study designed and tested a mixed intervention program of both MT and DMT through a 3-month intervention and training for children with ASD in the family setting including parent and child. A pre-test and post-test were conducted in the experimental and control groups, and the childhood autism rating scale (CARS) and autism treatment evaluation checklist (ATEC) scales were used to assess the severity of ASD symptoms and the effects of intervention. A *t*-test and analysis of variance were performed based on the experimental results. The results indicated that the experimental and control groups did not differ significantly on the CARS pre-test (*t* = 1.218, *p* > 0.05) and that there was no significant difference in the ATEC pre-test (*t* = 0.546, *p* > 0.05; *F* = 0.074, *p* > 0.05, partial η^2^ = 0.003). There was no significant difference between the pre- and post-test scores for the CARS in the control group (*t* = 0.635, *p* > 0.05), and there was no significant difference between the pre- and post-test scores for the ATEC in the control group (*t* = 0.027, *p* > 0.05; *F* = 5.251, *p* > 0.05, partial η^2^ = 0.313). There was a significant difference between the pre- and post-test scores on the CARS in the experimental group (*t* = 4.327, *p* > 0.05) and the pre- and post-test scores on the ATEC in the experimental group (*t* = 5.763, *p* > 0.01; *F* = 32.615, *p* > 0.01, partial η^2^ = 0.759), with the post-test scores being lower than the pre-test scores. This demonstrates that the mixed intervention of MT and DMT in the family parent-child setting can reduce autism and improve social communication impairment in children with ASD.

## Introduction

In 2003, the US Centers for Disease Control and Prevention estimated that 1 in 68 (1.47%) American children was affected by autism. In 2019, the ASD monitoring network (ADDM) reported that the prevalence of autism in American children was 1 in 59 (1.69%), while the prevalence of ASD in British and Dutch children was 1 and 0.6–0.7%, respectively. The second national census of the disabled in 2006 revealed that people with ASD constituted 36.9% of the disabled population in China, with an estimated 40,000 children suffering from ASD-related mental disabilities. According to a report published in 2014, the number of patients with ASD in China has surpassed 10 million, and the number of children under the age of 14 has reached more than 2 million, with an annual growth rate of more than 200,000. According to the statistics presented above, there is higher prevalence of ASD among children than cancer, diabetes, and AIDS. ASD has become the “number one killer” for children with mental disorders worldwide.

Currently, there is no cure for autism. Families of autistic patients must send their loved ones to rehabilitation training institutions. At present, half of the children with ASD in China who require rehabilitation training are still waiting in line. Consequently, nearly one-fifth of them miss out on the optimal time for rehabilitation training. Many children with ASD delay rehabilitation training until they are nearly 7 years old, which is extremely detrimental to their early recovery. The treatment cost for children with ASD is also very high, and long-term rehabilitation program is time-consuming. Furthermore, parents of children with ASD lack the necessary professional knowledge and are unable to devote enough time to continuous rehabilitation training for their children, which delays the opportunity for early treatment and greatly diminishes the therapeutic effect. Therefore, it is crucial to develop a feasible and effective rehabilitation treatment model for children with ASD in family settings.

Numerous methods are available for rehabilitating children with ASD. According to domestic and international research, music rehabilitation training therapy (MT) and dance movement rehabilitation training therapy (DMT) are the most commonly used rehabilitation training therapies in the family setting. Existing research indicates that both approaches have therapeutic benefits for children with ASD. However, previous research has frequently focused on the intervention effect of a particular therapy, and rehabilitation training conducted in specialized training institutions. Social impairment is the most significant core deficit across all dimensions of autism symptoms, and improvement in social impairment is of great value for children with ASD. This study aimed to combine music therapy and dance movement therapy to provide rehabilitation training for children with ASD in family settings, investigate the effect of a mixed treatment mode on the improvement of ASD children’s social impairment, and promote the improvement of their social ability and quality of life. This study will also attempt to establish and promote a mixed music and dance rehabilitation training model for the children’s families. In addition, this study will seek to establish and promote a mixed rehabilitation training mode of music and dance in children with ASD.

## Theoretical basis

### Autism spectrum disorder

In 2013, the Diagnostic and Statistical Manual of Mental Disorders (DSM-5) declared autism spectrum disorder (ASD) as a condition originating in childhood. However, the underlying cause remains unknown; studies have attributed genetics, infections, and immunity as well as physical and chemical factors during pregnancy as possible causes. There is higher prevalence of ASD among boys than girls, with a ratio of 4:1.

In children, the clinical symptoms of ASD include social dysfunction, verbal communication disorder, interest and behavior disorder, abnormal perception disorder, and intellectual and cognitive deficits. Over the past few decades, researchers have developed several rehabilitation training and treatment methods for children with ASD. Methods include structured teaching methods, sensory integration training methods, applied behavior analysis (ABA), interpersonal relationship development intervention therapy (RDI), painting therapy, psychological sitcom therapy, music therapy (MT), and DMT. MT has the ability to transcend language, act directly on people’s emotions, reach the underlying layer of the soul, project to the heart, and release calming emotions. DMT is the integration of a person’s physical, emotional, and cognitive processes into an expression of a self-directed life. These two treatment approaches have distinct benefits and therapeutic effects on children with ASD.

### Social communication impairments

Social communication impairment (SCI) is one of the primary symptoms and diagnostic criteria for autism. According to Rutter and Lockyer (1967), Schuler and Prizant (1985); Leslie (1987), and Tager-Flusberg (1993), children with ASD have significant social cognitive impairment; Kanner (1943); Hobson (1992), Kasari et al. (1993), and Loveland et al. (1994) found that children with ASD have social-emotional disorders. Social cognitive impairment, socioemotional impairment, and lack of social skills result in children with ASD having severe problems in social interaction, as evidenced by a lack of social gaze, lack of attachment to family members, and inability to form relationships with peers. Additionally, they have difficulty playing games normally, maintaining social interactions, and interpreting verbal and non-verbal cues, emotions, and facial expressions.

Methods such as applied behavior analysis, social story, social game, and music therapy are frequently used to intervene and treat social impairment in children with ASD, both domestically and internationally. Each of these methods has a therapeutic effect. No relevant research has been conducted on the effect of combining music therapy and dance movement therapy on social impairment in children with ASD.

### Music therapy

Music therapy for the treatment of physical and mental illnesses has existed since antiquity; however, its name and form were not determined until the early twentieth century. Music therapy is a systematic form of intervention. In this process, therapists use various forms of music experience and the therapeutic relationship developed throughout the treatment process as a driving force to assist the treated object in achieving its health goals. Long-term research has been conducted on the effects of music therapy on language and communication skills, social skills, self-confidence, and emotional experiences. Many clinical studies have demonstrated that music therapy has a positive effect on these areas. Moreover, children with autism have a strong interest in and response to music. Compared to other rehabilitation treatments, music therapy elicits greater enthusiasm and participation from children with autism. Cao (2009), for instance, conducted a case study on the social interactions of children with autism using improvisational music therapy. The results demonstrated that improvisational music therapy improved the children’s social interaction skills and had a positive effect on their musical abilities. In addition, it has a positive impact on the reduction of problem behaviors and strong social validity. Wang (2010) used music to treat children with autism and achieved remarkable results, and Stavropoulos and Carver (2018) discovered that music therapy altered the connection between different brain regions in children with ASD and improved their behavior and social relationships. FMRI, DTI, and other imaging studies demonstrate that through singing, rhythm recitation, musical instrument performance, and other methods, the brain is activated, brain plasticity is induced, and children’s linguistic ability is fostered and developed during music therapy activities.

### Dance movement therapy

This study focused on interventions for children with autism, whose body movements were distinctive. Given that DMT is characterized by improvisation, creativity, and aesthetics, it is distinct from other body-based somatic psychotherapies. Through physical movement, DMT can improve the symptoms of physical and mental behavioral disorders in children with ASD and can be used to repair or improve obstacles within the movement field so that children can achieve healthy behaviors. In the 1960s, the American therapist Bethkalish Weiss began researching the psychodynamic personality and motor behavior of autistic children and became a pioneer in this field. Goodill (2005) described the creative contribution of Nicholas Kasovac, a dance therapist, who integrated movement analysis and the concepts of Kesternberg Movement Profile (KMP) into DMT techniques. Based on this, Kasovac created a healing environment that is both physically and mentally safe in motion communication. Scharoun et al. (2014) discussed the use of DMT as a group intervention for autism and found that group cohesion improved, while aggression decreased. Hildebrandt et al. (2016) conducted a 2-year study on the effects of DMT on the negative symptoms (social deficits) of autistic patients and found that DMT can effectively reduce the negative symptoms of ASD, thereby enhancing their social skills. Nelson et al. (2002) utilized a multi-baseline intervention design to encourage children with ASD to engage in creative circle dancing. The results demonstrated that the intervention improved their social awareness. Pang (2009) and Li (2016) reviewed research-related theories and methods of DMT interventions in children with autism. Lin (2014) and Ren (2014) investigated the efficacy of DMT in the treatment of special children and discovered that children with mental retardation benefit significantly from DMT by improving their social communication skills while reducing hyperactivity and disorganized behavior and have a positive follow-up effect on their behavioral problems, but do not benefit significantly from DMT in terms of improving their conduct problems. Magulanmu (2020) examined the intervention theory and methods of DMT on children with autism and conducted empirical research. He concluded that DMT facilitates the physical development of children with autism and promotes interpersonal communication among them.

### Parent-child family situation

Within the family setting, parents conduct the MT and DMT intervention training for their autistic children. This environment differs from the external environment provided by society and schools. Thompson (2014) conducted a family centered music therapy intervention with eleven autistic children aged 3–6 years. The results indicated that parents of autistic children integrated music therapy activities into their families, which went beyond the direct involvement of music therapists and contributed to the long-term effectiveness of music therapy. Taiwanese dance therapists Zhu et al. (2015), along with others, collaborated on DMT application research for children with ASD. Parent-child DMT intervention for autistic children demonstrated the beneficial effects of body rhythm. Kim et al. (2016) also highlighted the need for music therapy for autistic children in families.

In conclusion, prior literature demonstrates that both MT and DMT have positive therapeutic effects on children with ASD, but the majority of existing studies are case studies, and quantitative studies are minimal. Although numerous studies have investigated the effect of family situation on the efficacy of treatment for children with ASD, the combination of MT and DMT in the family setting has not been examined.

## Intervention program

### Theoretical basis of program design

#### Embodiment theory

Embodied theory is an interdisciplinary theoretical method that affords more opportunities for empirical development and research, and is applicable to the field of art therapy, including DMT. Embodied theory primarily integrates views based on the body and people’s views of the body. It is founded on phenomenological comprehension of the role of the body and its sense of movement. Embodied theory emphasizes the role of the body in cognitive processes and the influence of physical touch on judgment. The dance movement design allows autistic children to make contact with their own bodies, their parents, and perform other activities while simultaneously forming correct cognition. The environment can be used to reduce cognitive load according to embodied theory. This study designed and arranged a parent-child home environment to provide a relaxed and enjoyable activity space for children with ASD, reduce the cognitive burden of children with ASD, and encourage the development of their social interests and social skills.

#### Interaction analysis theory

Bourne’s interaction analysis theory states that people’s psychological states are divided into three: parental self-state (parental state for short), adult self-state (adult state for short), and child self-state (child state for short). This state is attainable by anyone, and each individual’s unique thoughts and behaviors are related to this state; for instance, demandingness stems from the parental state, while rationality and dependence stem from the adult and child states, respectively. For those who are not sociable, socially phobic, not adept at thinking, and self-isolating, their hyperactive parental self-state is the cause, and they may benefit from music therapy, which allows the power of music to stimulate the child self-state, and allows the patient to experience and activate the state through musical activities.

#### Humanistic theory

Based on growth motivation, creative music therapy helps children develop intrinsic motivation to enrich and affirm themselves, in accordance with the humanistic theory. It is the most effective therapy experience to achieve integration, ego identity, integration with others, and integration with the world.

### The specific content of the program

A dance movements and music mixed intervention program for children with ASD was developed, including body cognition, breathing training, body movements, and interactive movements. A therapist acted as the mother, and a typical child acted as the child with ASD. The content of the intervention was demonstrated and video recorded. Specifically, the following were covered in the intervention training.

#### Body awareness

Body awareness involves using hands to touch various parts of the body, such as the head, shoulders, chest, waist, feet, knees, and hands, as instructed. Touching occurs from top to bottom, and from bottom to top. During the process, music and instructions from “My Body” was played. The training duration was 10 min.

#### Breathing training

This training consists of two components, inhalation and expiration. The knees are brought together, the buttocks rest on the calf, the upper body is kept upright, the body is in a relaxed state, and the prescribed breathing exercises are performed. Background music and instructions from “Breathing Song” were played. The training duration was 10 min.

#### Body movements

(1) Upper-body movement: The student should move their upper body but not lower body. They follow instructions and perform actions such as shaking hands, raising hands, and holding hands akimbo in sequence; playing background music “Robot Walk” with instructions; the duration of training was 8 min.

(2) Lower body movements: The student should not move the upper limbs; instead, they move the lower limbs and perform the duck step, squat, and lunge jump in sequence; the song “Let It Go” was played with instructions; the duration of training was 10 min.

(3) Left and right ipsilateral movements: The students perform ipsilateral walking with left and right movements; playing the background music “Let It Go”; the duration of training was 10 min.

(4) Whole body coordinated action: The students walk, ride on horseback, tiptoe, skate, and dance, and jump on one foot to the rhythm and instructions of the music; “Listen and Move” was played as background music; the duration of training was 10 min.

#### Interactive action

(1) Dot Palms: parents massage and apply compression to the child’s palms, fingers, feet, knees and other body parts in accordance with the rhythm and speed of the music; “Dip Palms” was played as background music; the duration of the training was 10 min.

(2) In Puppet Adventure: parents and children act together, parents stand and relax, and children manipulate their parents’ limbs to create various body movements. Parents must hold the position until the child consents to its release; “Waltz Music” was played; the duration of the training was 10 min.

(3) Clapping Dance: parents and children sit facing each other on the ground and clap hands to music; “Clap Song” was played during this time; the duration of the training was 10 min.

(4) Two-person top cow: parents and children lie face-to-face on the ground and grapple with their heads. Parents should pay attention to their children’s strength and protect them from harm; “Top Cow Nursery Rhymes” was played as background music; the duration of the training was 10 min.

(5) Train driving task: parents and children perform the task where children are the lead locomotives. The parents put their hands on their children’s shoulders to act as a train car and walk around the site; “little train” was played in the background; the duration of the training was 10 min.

### Intervention course schedule

The intervention program consisted of twelve 40-min sessions. The first 10 min consisted of breathing training, followed by 30 min of three modules focusing on body cognition, body movement, and interactive movement training. The arrangements and contents of the last three modules were randomly combined. Children with ASD received intervention training at home every Saturday from 10:00 to 10:40 am for 3 months.

### Parents training

Parents are the hosts, participants, and recorders of the intervention training for the children. To ensure the experimental effect, the researchers organized a 1-week online training program for the parents of the experimental group to familiarize them with the course content and teach them intervention methods, behavior observation, and recording methods. Following the training, parents’ businesses were evaluated. Only individuals who passed the evaluation were included in the experiment. Parents were required to participate in weekly online meetings before and after the intervention to discuss precautions and experiences.

## Research methods and processes

### Experimental design

In this study, a “pre-test and post-test group design” was adopted, and 30 children with ASD were randomly divided into an experimental group and a control group. The parents of the experimental group participated in the researcher-organized training, watched the intervention course video with their children with ASD, and led their children in MT and DMT mixed training activities. Children with ASD in the control group did not receive training in the intervention course and lived and studied normally. Neither the experimental group nor the control group was permitted to take drugs or engage in other therapeutic activities during the experiment.

### Research tools and equipment

#### Childhood autism rating scale

The childhood autism rating scale (CARS) was developed in 1980 by American psychologists Schopler, Reichler, and Renner. It is a standardized scale for distinguishing and diagnosing autism in children based on 15 dimensions, including interpersonal relationships, adaptability, sensory and perceptual responses, and emotional and behavioral responses. In this study, the CARS was primarily used for subject screening and for comparing pre- and post-test scores. Parents scored the autism status of children with ASD online, and the children were required to complete a pre- and post-intervention assessment. This study adopted Li Xuerong’s revised version of CARS. According to the test results of Yin Qingyun et al. at a mental hospital in Guangzhou, the Cronbach’s coefficient of the 15 CARS items was 0.781 and their MIC was 0.324. The correlation coefficients between the majority of the items were significant at *p* < 0.05, and the CARS reliability was high. Cronbach’s coefficients for the five main dimensions ranged from 0.396 to 0.639, indicating the scale’s high internal consistency.

#### Autism treatment evaluation checklist

Autism treatment evaluation checklist (ATEC) refers to the dynamic evaluation of treatment effectiveness. The primary objective was to compare the children’s autism symptoms before and after the intervention. The scale consists of 77 items, divided into four sections: expressive/verbal communication (14 items), social ability (20 items), perception/cognitive ability (18 items), and health/physiology/behavior (25 items). The ATEC scale is a widely used professional evaluation scale. Filling out the scale does not require specialized knowledge, making it convenient for the parents. The scale’s high operability and widely acknowledged evaluation effect contributed to the scientific credibility of the experiment. Parents completed pre- and post-tests on the ATEC scale online. Given that this study aimed to investigate the therapeutic effect of MT and DMT mixed interventions on social impairment in children with ASD, the ATEC score refers to the ATEC social ability subscale score without special instructions. According to the 2004 test results of Lu Jianpin, Yang Zhiwei, Shu Mingyao, Su Linyan, and others, ATEC has high reliability and validity and is suitable for pre- and post-intervention comparative analyses.

#### Hardware equipment

Parents require a TV set to play the instructional video and music of the treatment; they should also possess a mobile phone with video recording capability to record the entire process of rehabilitation training for children with ASD.

### Research methods

#### Measurement methods

The CARS was used to assess the severity and developmental status of children with ASD, and the ATEC scale was used to assess the changes in the social status of children with ASD before and after intervention.

#### Experimental method

Dance movements and music therapy activities were conducted for twelve families. The effects of MT and DMT mixed therapy were verified during a period of 3 months of actual intervention.

### Research subjects

The subjects in this study were recruited *via* the Internet, and applicants were required to provide ASD diagnosis certificates from the hospital’s psychiatry department. A total of 52 children with ASD participated in the registration, and all participants were screened and confirmed using the CARS. Thirty children with ASD were selected, including 24 boys and 6 girls. Thirty children with ASD did not have any other illness. All participants’ parents signed the informed consent form, agreeing to participate in the required training, complete the training records, and collaborate with the researchers to complete the various tasks.

### Research hypotheses

Based on the evidence collected in the theoretical basis section, the following research hypotheses are proposed:

(1) The pre- and post-test scores for CARS and ATEC were significantly different in the experimental group; The mixed intervention of MT and DMT in family parent-child situations significantly improved ASD children’s symptoms of autism and social impairment;

(2) The CARS and ATEC scores did not differ significantly between the pre- and post-tests in the control group.

## Data analysis

To test the research hypothesis, SPSS26.0 was used to analyze the data.

### Descriptive statistical analysis of subjects

As shown in Table 1, the gender distribution of the participants in the experiment was uneven, with 80% male and 20% female. This difference may be due to sex differences in the prevalence of ASD. The percentage of children with mild to moderate ASD was 76.67%, while those with severe ASD was 23.33%. The percentage of 5–6 year olds and 7–8 year olds were 53.33 and 46.67%, respectively, which were almost equally distributed.

**TABLE 1:** Descriptive statistical analysis results.

Variable	Category	Count	Percentage
Gender	Male	24	80.00%
	Female	6	20.00%
Severity of ASD symptoms	Mild	11	36.67%
	Moderate	12	40.00%
	Severe	7	23.33%
Age group	5–6	16	53.33%
	7–8	14	46.67%

### Comparison of pre-test scores between experimental group and control group

Based on the results of the Levine variance equivalence test in Table 2, it can be seen that the variances of the CARS and ATEC pre-test scores of the experimental group and the control group are homogeneous. An independent-samples *t*-test was used to test the CARS pre-test scores of the two groups. *p* > 0.05 indicated no significant difference in the CARS pre-test scores between the experimental and control groups. Using the independent samples *t*-test (*p* > 0.05), the ATEC pre-test scores were compared between the experimental and control groups. The ATEC pretest scores of the experimental and control groups did not differ significantly. The results of the test indicated that the ASD children in both the experimental and control groups had the same level of autism before the training and that both the experimental and control groups were homogeneous groups.

**TABLE 2:** Independent sample *t*-test results of CARS and ATEC pre-test scores in experimental group and control group.

Scale	Experimental group		Control group		t	p
	Average value	Standard deviation	Average value	Standard deviation		
CARS	34.63	1.035	33.51	1.243	1.218	0.247
ATEC	38.32	3.743	37.83	4.670	0.546	0.621

### Comparison of pre- and post-test scores between the experimental group and the control group

Paired-sample *t*-tests were conducted on the pre- and post-test scores for CARS and ATEC in the experimental group. AS shown in Table 3, the pre- and post-test scores varied significantly, and the CARS and ATEC post-test scores of children with ASD were significantly lower than the pre-test scores. Thus, Hypothesis 1 is supported.

**TABLE 3:** Paired sample *t*-test results for the pre- and post-test scores of CARS and ATEC in the experimental group and the control group.

Group	Scale	Pre-test		Post-test		t	p
		Average value	Standard deviation	Average value	Standard deviation		
Experimental group	CARS	34.63	1.035	29.45	1.137	4.327	0.035*
	ATEC	38.32	3.743	32.56	3.863	5.763*	0.007**
Control group	CARS	33.51	1.243	32.81	0.973	0.635	0.692
	ATEC	37.83	4.670	37.80	2.432	0.027	0.712

*p ≤ 0.05, **p ≤ 0.01.

On the pre- and post-test scores of the CARS and ATEC in the control group, the paired sample *t*-test revealed no significant difference between the two scores, suggesting that there was no significant improvement in the autism symptoms of children without intervention training. Thus, hypothesis 2 holds.

### Comparison of post-test scores between the experimental group and the control group

The results of the Levine variance equivalence test indicated that the variances of the CARS and ATEC post-test scores of the experimental and control groups were homogeneous. CARS and ATEC post-test scores were compared between the experimental and control groups using independent sample *t*-tests (*p* < 0.05). It can be seen from Table 4, there was a significant difference in the post test scores of the 729 CARS and ATEC between the experimental and control groups. Both the CARS and ATEC scores of the experimental group given the intervention were lower than those of the control group, indicating that the mixed treatment of MT and DMT had a significant effect on the experimental group.

**TABLE 4:** Independent sample *t*-test results of CARS and ATEC post-test scores in experimental group and control group.

Scale	Experimental group		Control group		t	p
	Average value	Standard deviation	Average value	Standard deviation		
CARS	29.45	1.137	32.81	0.973	−2.942	0.047*
ATEC	32.56	3.863	37.80	2.432	−4.362	0.006**

*p ≤ 0.05, **p ≤ 0.01.

### ANOVA for autism treatment evaluation checklist scores

As shown in Table 5, the *p*-values for the ATEC scores of both the experimental 740 and control groups were greater than 0.05, indicating that the ATEC scores of the two groups were in line with the normal distribution, and a repeated measures analysis of variance could be performed.

**TABLE 5:** Shapiro–Wilke (S-W) test results.

	Group	Statistics	Def	Sig.
Pre-test score	Experimental group	0.947	15	0.687
	Control group	0.958	15	0.762
Post-test score	Experimental group	0.951	15	0.674
	Control group	0.972	15	0.932

Machly’s *W* = 0.673, *p* < 0.05, did not meet the sphericity test in Table 6. As a result, in the event of correction, the results from the multivariate test prevail. The results in Table 7 indicate that the main effect of the number of measurements is *F* = 31.245, *p* < 0.001, and partial η^2^ = 0.759, indicating a significant impact of the number of measurements. In addition, the interaction between the number of measurements and group was *F* = 6.147, *p* = 0.008, and partial η^2^ = 0.354, indicating that there was also a significant interaction between the number of measurements and the group.

**TABLE 6:** Mauchly’s test of sphericity.

Within subjects effect	Mauchly’s W	Approx. chi-square	df	Sig.	Epsilonb		
					Greenhouse- geisser	Huynh-feldt	Lower-bound
Number of measurements	0.673	7.823	1	0.021	0.762	0.856	0.500

a. Design: Intercept + Group; Design within the main body: Number of measurements.

b. This can be used to adjust the degrees of freedom used in the average tests of significance. Tests of within-subject effects displayed the correct tests.

**TABLE 7:** Multivariate test.

Effect	Group	Value	F	Hypothesis df	Error df	Sig.	Partial η^2^
Measurement number	Bilet Trajectory	0.728	31.245b	1	28	0.000	0.759
	Wilk’s Lambda	0.252	31.245b	1	28	0.000	0.759
	Hotelling’s Trajectory	2.935	31.245b	1	28	0.000	0.759
	Roy’s Largest Root	2.935	31.245b	1	28	0.000	0.759
Measurement number*group	Pillai’s Trajectory	0.368	6.147 b	1	28	0.007	0.354
	Wilk’s Lambda	0.642	6.147 b	1	28	0.007	0.354
	Hotelling’s Trajectory	0.573	6.147 b	1	28	0.007	0.354
	Roy’s Largest Root	0.573	6.147 b	1	28	0.007	0.354

a. Design: Intercept + Group; Design within the main body: number of measurements.

b. Exact Statistics.

**TABLE 8:** Repeated measures ANOVA results for ATEC scores.

	Pre-test $\bar{\text{x}}$ ± std	Post-test $\bar{\text{x}}$ ± std	Repeated measures F test		
			F	Sig.	Partial η^2^
Experimental group	38.32 ± 3.743	32.56 ± 3.863			
Control group	37.83 ± 4.670	37.80 ± 2.432			
Group main effects			2.05	0.17	0.09
Measure number main effects			51.56	<0.001**	0.70
Group * Measure number			9.63	0.001**	0.30

*p ≤ 0.05, **p ≤ 0.01.

In the repeated-measures analysis of variance of ATEC scores, the main effect of group was not significant (*p* > 0.05), but the main effect of the number of measurements and the interaction were extremely significant (*p* < 0.001).

As shown in Table 9, there was no significant difference between the ATEC pre-test score and control groups (*F* = 0.074, *p* > 0.05, partial η^2^ = 0.003). In the post test, the simple effect of group was significant (*f* = 6.531 < 0.05, partial η^2^ = 0.229). The results of the *t*-test supported this conclusion. The results indicated that the experimental and control groups were homogeneous.

**TABLE 9:** Simple effect analysis table of ATEC score groups.

Measure Number		Sum of Squares	df	Mean Square	F	Sig.	Partial η^2^
Pre-test	Compare	0.070	1	0.070	0.074	0.788	0.003
	Error	20.891	28	0.950			
Post-test	Compare	12.470	1	12.470	6.531	0.018*	0.229
	Error	42.009	28	1.91			

*p ≤ 0.05, **p ≤ 0.01.

As shown in Table 10, the simple effect of the number of measurements in the experimental group was highly significant (*p* < 0.001). The simple effect of the number of measurements in the control group was not statistically significant (*p* > 0.05). In line with the results of the *t*-test, the intervention of the experimental group significantly affected the results of ATEC, and Hypotheses 1 and 2 were proved again.

**TABLE 10:** Simple effect analysis table of ATEC score measure times.

Group		Value	F	Hypothesis df	Error df	Sig.	Partial η^2^
Experimental group	Bilet Trajectory	0.721	32.615*^a^*	1	28	0.000	0.759
	Wilk’s Lambda	0.246	32.615*^a^*	1	28	0.000	0.759
	Hotelling’s Trajectory	3.125	32.615*^a^*	1	28	0.000	0.759
	Roy’s Largest Root	3.125	32.615*^a^*	1	28	0.000	0.759
Control group	Pillai’s Trajectory	0.314	5.251*^a^*	1	28	0.066	0.313
	Wilk’s Lambda	0.639	5.251*^a^*	1	28	0.066	0.313
	Hotelling’s Trajectory	0.514	5.251*^a^*	1	28	0.066	0.313
	Roy’s Largest Root	0.514	5.251*^a^*	1	28	0.066	0.313

a. Exact Statistics.

## Discussion

An intervention program designed by this study using MT and DMT for children with ASD was developed for a 3-month training and intervention based on family parent-child interactions. The experimental data revealed that the mixed intervention of MT and DMT based on the parent-child relationship in the family reduced the autism symptoms of children with ASD and improved their social impairment. The mixed intervention of MT and DMT creates a pleasant activity space that is conducive to the cognitive rules and interests of children. Children with ASD respond spontaneously to music and dancing. The mixed intervention has led to the reduction of autism symptoms and the improvement of social impairment. Second, the mixed intervention training used music and dance movements to replace the repetitive, stereotypical speech and behavior patterns of children with ASD, and the conversation between parents and children has also led to the development of social interests and skills. Through the use of music and physical movements, children with ASD were able to release repressed emotions and generate positive emotional experiences. Children with ASD gain self-confidence and see improvements in their autism symptoms and social impairment following successful participation in the activities.

## Conclusion

In view of the obvious social impairment in children with ASD, existing research focuses on the effects of individual MT or DMT interventions, and few studies have examined the intervention of social impairments in children with ASD under the family parent-child system. Therefore, this study designed a mixed intervention scheme of MT and DMT, and conducted a 3-month intervention and training program for children with ASD within a family setting. Based on the pre- and post-test experimental designs for the experimental and control groups, CARS and ATEC were used to evaluate the severity of ASD symptoms and the effects of the intervention, and *t*-tests and analysis of variance were performed on the experimental results. The results of the experiment indicated that there were no significant differences in pre-test scores of CARS and ATEC between the experimental and control groups, and both groups were comparable before the intervention. Both the CARS and ATEC pre- and post-test scores were not significantly different in the control group, whereas the pre- and post-test scores of the experimental group were significantly different, and the post-test scores were lower than the pre-test scores. The mixed intervention of MT and DMT in a family setting has been shown to reduce the severity of autism in children with ASD as well as improve their social impairment. The results of this study are consistent with the strong positive effects of only MT or DMT interventions on children with ASD in the family environment at home and abroad. The strong significance of MT and DMT mixed intervention on the parent-child relationship is demonstrated through the experiment, which provides evidence for the effectiveness of parent-child intervention for children with ASD in the family environment. It also provides the basis for a complete intervention program with useful promotion and application effects.

The following limitations were apparent in this study: First, there is a clear sex difference in the incidence of ASD among children. As evident, the number of male participants greatly exceeded females. Second, the duration of the intervention training was 3 months, and the period of improvement in ASD symptoms was longer. This may have affected the experimental results. Therefore, future interventions and training should be implemented. Third, the content of the intervention should be expanded and optimized for a wider age group as the age group of the children in this study was limited to a narrow range of 5–8 years.

## Data availability statement

The original contributions presented in this study are included in the article/supplementary material, further inquiries can be directed to the corresponding author.

## Ethics statement

Ethical review and approval was not required for the study on human participants in accordance with the local legislation and institutional requirements. Written informed consent from the patients/participants was not required to participate in this study in accordance with the national legislation and the institutional requirements.

## Author contributions

HR: writing – original draft. GR, YZ, and ZJ: supervision. All authors have contributed equally to this work.
